# Quantifying *Batrachochytrium dendrobatidis* and *Batrachochytrium salamandrivorans* Viability

**DOI:** 10.1007/s10393-019-01414-6

**Published:** 2019-05-23

**Authors:** Alexa Lindauer, Tiffany May, Gabriela Rios-Sotelo, Ciara Sheets, Jamie Voyles

**Affiliations:** Department of Biology, University of Nevada, Reno, 1664 North Virginia Street, Reno, NV 89557 USA

**Keywords:** *Batrachochytrium dendrobatidis*, *Batrachochytrium salamandrivorans*, MTT assay, Pathogen, Disease, Amphibian declines

## Abstract

The disease chytridiomycosis is responsible for global amphibian declines. Chytridiomycosis is caused by *Batrachochytrium dendrobatidis* (*Bd*) and *B. salamandrivorans* (*Bsal*), fungal pathogens with stationary and transmissible life stages. Establishing methods that quantify growth and survival of both life stages can facilitate research on the pathophysiology and disease ecology of these pathogens. We tested the efficacy of the MTT assay, a colorimetric test of cell viability, and found it to be a reliable method for quantifying the viability of *Bd* and *Bsal* stationary life stages. This method can provide insights into these pathogens’ growth and reproduction to improve our understanding of chytridiomycosis.

Chytridiomycosis is an amphibian disease caused by the fungal pathogens *Batrachochytrium dendrobatidis* (*Bd*) and *B. salamandrivorans* (*Bsal*; Berger et al. 1998; Longcore et al. 1999; Martel et al. 2013). Both pathogens have caused amphibian declines and are considered threats to biodiversity (Skerratt et al. 2007; Wake and Vredenburg 2008; Stegen et al. 2017). Although the pathogenesis of *Bsal* is less understood (Van Rooij et al. 2015), development of lethal chytridiomycosis from *Bd* has been linked with increases in infection intensity (i.e., *Bd* loads; Voyles et al. 2009; Vredenburg et al. 2010). As such, investigations on *Bd* and *Bsal* growth have been key to understanding the biology of this disease (Woodhams et al. 2008; Voyles et al. 2017).

Both *Bd* and *Bsal* have complex life histories (Longcore et al. 1999; Martel et al. 2013). Motile *Bd* and *Bsal* zoospores encyst and develop into zoosporangia. Stationary zoosporangia produce zoospores and release them into the environment or back onto the host (Longcore et al. 1999; Berger et al. 2005; Martel et al. 2013). Since increases in zoospore production are not always proportional to increases in zoosporangia growth rate (e.g., at temperatures below the *Bd* thermal optimum; Woodhams et al. 2008; Voyles et al. 2012), understanding differences in growth and reproduction of specific life stages is important to understand the infectivity of these pathogens and the trade-offs they face under different conditions.

Multiple methods have been used to measure *Bd* and *Bsal* growth in vitro (Piotrowski et al. 2004; Martel et al. 2013). Zoospore production can be measured by counting motile zoospores using a hemocytometer, and stains (e.g., trypan blue, SYBR-14, propidium iodide) can improve count accuracy (Stockell et al. 2010; McMahon and Rohr 2014). Lag, exponential, and stationary phases of *Bd* and *Bsal* growth can be measured by reading optical density (OD) at 490 nm (Rollins-Smith et al. 2002, Piotrowski et al. 2004; Rollins-Smith et al. 2006). However, OD measurements lack specificity because they do not differentiate between living and dead cells.

We tested the efficacy of an MTT assay in measuring *Bd* and *Bsal* growth and viability. The MTT assay is a reliable colorimetric test for cell viability that has been used in unicellular fungi and mammalian cell lines (Levitz and Diamond 1985; Freimoser et al. 1999). MTT (3-(4,5-dimethylthiazol-2-yl)-2,5-diphenyltetrazolium bromide) is a yellow tetrazolium salt that is reduced to purple MTT–formazan crystals in metabolically active cells (Mosmann 1983; Liu et al. 1997). The color change can be quantified by solubilizing the formazan crystals and reading culture absorbance at 570 nm, the most sensitive wavelength for this assay (Altman 1976).

We conducted experiments to (1) optimize MTT concentration and incubation time for *Bd*, (2) test the efficacy of the assay using serial dilution, and (3) apply the assay to quantify *Bd* and *Bsal* growth and viability over time. In addition, we measured zoospore production and zoosporangia growth by counting zoospores and reading OD_490_ to relate the MTT assay to other accepted quantification methods.

We revived *Bd* and *Bsal* isolates (*Bd* MYLF-16343, *Bd* NMBF-04, *Bsal* AMFP13/1) from cryopreservation (Boyle et al. 2003) and passaged them following established protocols (Longcore et al. 1999; Martel et al. 2013). Specifically, we cultured the pathogens in TGhL media in tissue culture flasks at 18°C for 7–9 days for *Bd* and at 10°C for 3–4 days for *Bsal* until we observed zoospore release. We then harvested zoospores by scraping cells from the flasks and filtering cultures through sterilized filter paper to remove zoosporangia and debris (Voyles 2011). We inoculated 100 μL diluted zoospore filtrate into 96-well plates (*Bd* concentration: 23 × 10^4^ zoospores/mL; *Bsal* concentration: 48 × 10^4^ zoospores/mL) and used heat-killed zoospore filtrate, heat-killed for 10 min in a 40°C water bath, as a negative control. We incubated the plates at temperatures within the pathogens’ optimal ranges (*Bd*: 17.5°C, *Bsal*: 10°C; Piotrowski et al. 2004; Martel et al. 2013).

To determine an optimum MTT concentration and incubation time, we added either 10 μL or 20 μL 5 mg/mL MTT in sterile PBS to 100 μL *Bd* culture and stopped the reaction after 30-min, 1-h, 2-h, or 24-h incubation (Mosmann 1983; Hansen et al. 1989). At each time point, we solubilized the formazan crystals by adding 140 μL sodium dodecyl sulfate in dimethylformamide solution (20% SDS/50% DMF w/v) and homogenizing gently (Hansen et al. 1989). We then measured OD at 570 nm (Biotek ELx800 Absorbance Reader). We fit asymptotic regression curves using the “nlme” package (Pinheiro et al. 2018) in R v3.4.3 (used for all analyses; R Core Team 2018). We corrected OD values by subtracting mean heat-killed OD from live well readings and compared incubation times and concentrations using *t* tests.

To test the efficacy of the MTT assay, we conducted a serial dilution experiment and measured *Bd* viability on the day of peak zoospore production. We inoculated 100 μL actively growing culture into sterile flat-bottom 96-well plates as described above and serially diluted the cultures in 50 μL TGhL media. We repeated the same dilution with heat-killed cultures as a negative control. We added 20 μL 5 mg/mL MTT, incubated for 2 h, solubilized the formazan product, and recorded OD at 570 nm. We fit a linear model to corrected OD_570_ to determine whether the MTT colorimetric signal was directly proportional to cell density.

To determine the viability of *Bd* and *Bsal* cultures over time, we used the MTT assay to quantify culture growth every other day for 12 days. On each sampling day, we used the optimized MTT assay (as described above) to measure viability in randomly selected wells. To compare the MTT assay to widely accepted methods for measuring *Bd* and *Bsal* growth and reproduction, we measured OD_490_ before initiating the MTT assay, and we quantified zoospore production using a hemocytometer. For *Bd* cultures, we compared OD with and without the addition of MTT over time using ANCOVA.

We found that MTT effectively stains *Bd* and *Bsal*, visibly staining viable zoosporangia purple. Asymptotic regression models (*P* < 0.001 for all parameters of both concentrations) show that OD_570_ readings of MTT-assayed cultures increased over 24 h, reached an asymptote after 4 h, and differed by MTT concentration (Fig. 1). For wells exposed to 20 μL 5 mg/mL MTT, we did not detect a significant difference between OD_570_ readings at 2 and 24 h (*t* test, *t*_(8)_ = − 1.84, *P* = 0.10). Wells incubated with 20 μL 5 mg/mL MTT had higher OD_570_ readings than wells incubated with 10 μL 5 mg/mL MTT (2 h: *t*_(8)_ = − 4.2687, *P* = 0.003; 24 h: *t*_(8)_ = − 4.1104, *P* = 0.003). We did not observe a reduction of MTT in purified zoospores on day 0 for either pathogen at these MTT concentrations.

**Figure 1:**
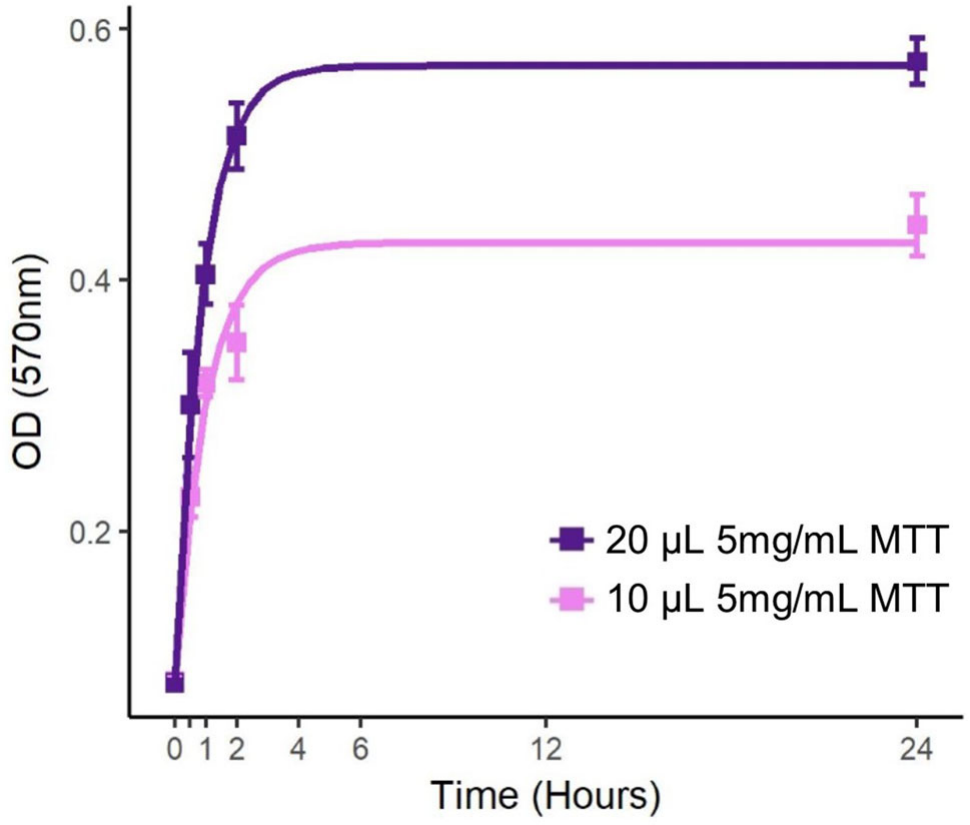
A comparison of optical density (OD) measurements at multiple incubation time points and two different concentrations of MTT (3-(4,5-dimethylthiazol-2-yl)-2,5-diphenyltetrazolium bromide) in wells containing *Batrachochytrium dendrobatidis* (*Bd*). The MTT assay provides a colorimetric measurement of metabolically active cells. A volume of 20 μL of 5 mg/mL MTT added to 100 μL of *Bd* culture (purple) produced a greater color change than 10 μL (pink). With the addition of 20 μL 5 mg/mL MTT, the assay was maximized after a 2–4-h incubation (Color figure online).

We found that OD readings of the MTT assay are directly proportional to *Bd* density (Fig. 2). OD readings increased linearly with increasing cell density for live *Bd* cultures assayed with MTT (*F*_(1,44)_ = 604.5, *P* < 0.001, *R*^2^ = 0.93).

**Figure 2:**
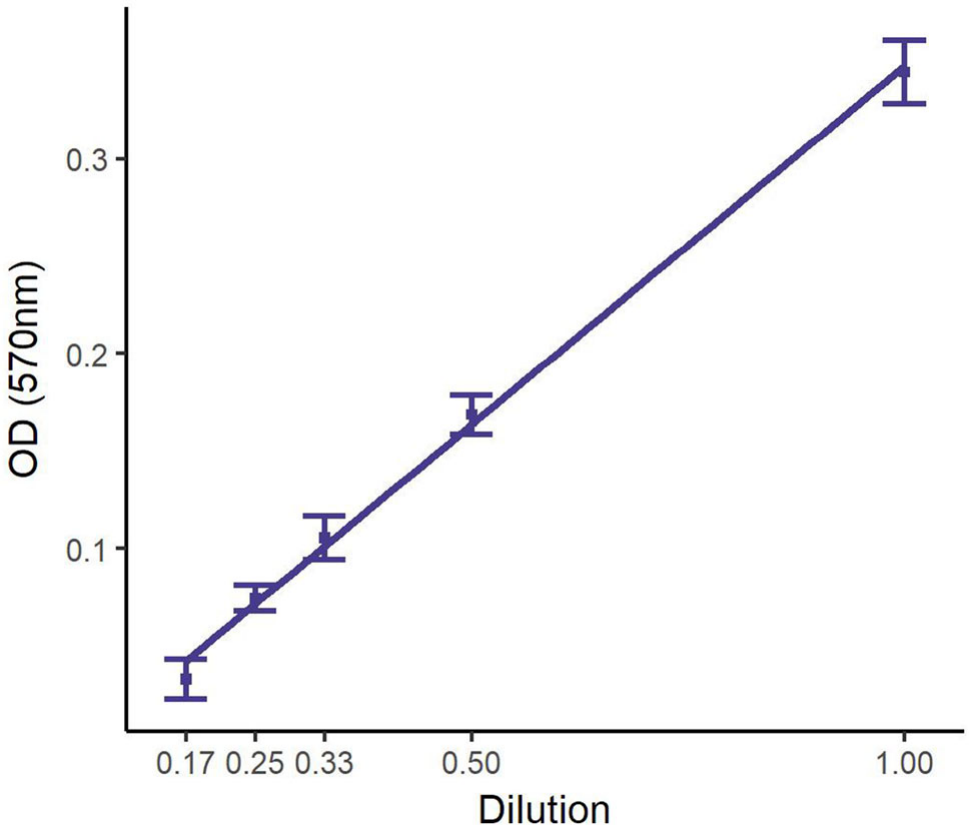
Optical density (OD) measurements in wells of serially diluted *Batrachochytrium dendrobatidis* (*Bd*). The wells containing *Bd* were incubated with 20 μL 5 mg/mL MTT for 2 h. OD readings were directly proportional to *Bd* densities.

We found that the MTT assay effectively measures *Bd* and *Bsal* viability over time. Reduction of MTT by live *Bsal* zoosporangia increased until peak zoospore release on day 8, after which zoosporangia viability decreased (Fig. 3). *Bd* zoosporangia viability increased through the period of peak zoospore release and plateaued on days 10–12 (Fig. 4). Culture growth as measured by OD_490_ without the MTT assay also increased over time and reached stationary phase by day 12. The MTT assay produced a higher colorimetric signal over time than OD_490_ measurements without MTT (Fig. 4; ANCOVA, *F*_(3108)_ = 360.4, *P* < 0.001; assay/day, *t* = 5.42, *P* < 0.001).

**Figure 3:**
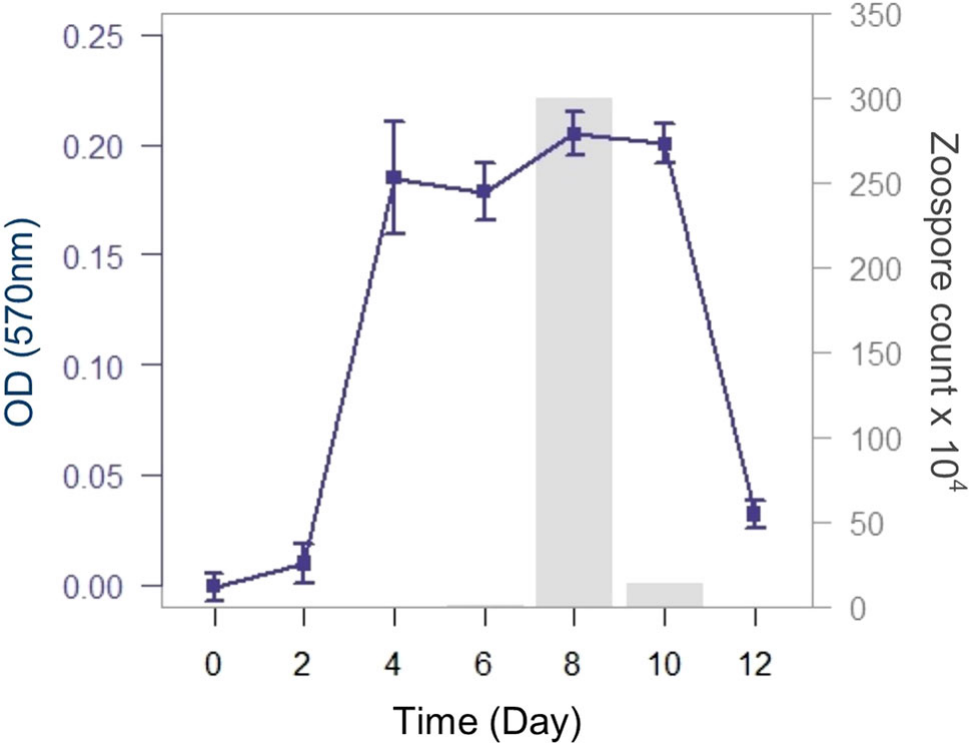
Growth of *Batrachochytrium salamandrivorans* (*Bsal*) over time using the MTT assay. Optical density measurements (OD_570_) were collected after incubating *Bsal* in 20 μL 5 mg/mL MTT (*n* = 12 randomly selected wells per day). The peak in OD readings coincided with maximum zoospore densities (gray bars) on day 8.

**Figure 4:**
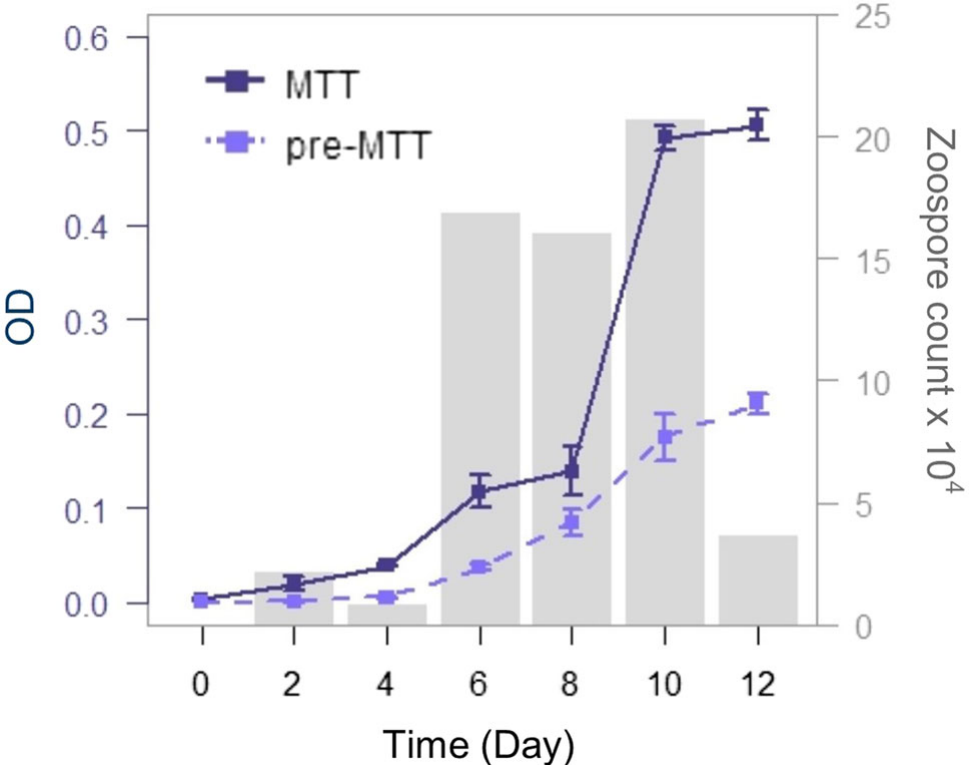
A comparison of optical density (OD) measurements of *Batrachochytrium dendrobatidis* (*Bd*) over time with and without incubation with MTT. OD measurements with MTT (solid dark blue, read at an optimum wavelength of 570 nm, *n* = 8 randomly selected wells per day) were greater than measurements without MTT (dashed light blue, read at an optimum wavelength of 490 nm). Maximum *Bd* growth was evident on days 10–12 and coincided with maximum zoospore densities on day 10 (gray bars) (Color figure online).

Our results suggest that the MTT assay is an effective tool for quantifying *Bd* and *Bsal* viability over time. Using a 2-h MTT incubation, the MTT assay is an efficient way to collect *Bd* and *Bsal* viability data during reproductive cycles. This method improves on measurements of OD_490_ alone because it can quantify growth of a specific life stage and it amplifies the OD signal (Fig. 4). Moreover, this method allows researchers to capture lag, exponential, stationary, and decay phases of pathogen growth (Fig. 3). When paired with measurements of zoospore production, this assay may help resolve other aspects of pathogen growth and reproduction.

The MTT assay will allow investigators to measure pathogen viability under ecologically relevant conditions, which can help improve understanding of pathogen growth in vivo. For example, amphibians contend with changes in ambient temperatures, which likely influences pathogen growth (Richards-Zawacki 2009; Rowley and Alford 2013). Using the MTT assay, pathogen growth and viability can be modeled in vitro to assess their responses to dynamic thermal environments (Woodhams et al. 2008; Voyles et al. 2012). In addition, MTT assays could provide an effective method for quantifying *Bd* or *Bsal* viability in the presence of inhibitory compounds such as antimicrobial peptides produced in frog skin glands, or antifungal metabolites produced by the amphibian skin microbiome (Rollins-Smith et al. 2002, 2006; Harris et al. 2009). Applying the MTT assay to a range of experimental *Bd* and *Bsal* research questions can help improve our understanding of the ecology of chytridiomycosis.
